# Herbal medicine for the management of idiopathic trigeminal neuralgia: A protocol for a systematic review of controlled trials

**DOI:** 10.1097/MD.0000000000020779

**Published:** 2020-06-19

**Authors:** Ji Hye Hwang, Jaseung Ku

**Affiliations:** a Department of Acupuncture and Moxibustion Medicine, College of Korean Medicine, Gachon University, Seongnam; b Bogwang Korean Medical Clinic, Seoul, Republic of Korea.

**Keywords:** herbal medicine, idiopathic trigeminal neuralgia, protocol, systematic review

## Abstract

**Background:**

Trigeminal neuralgia is an oral facial pain that is limited to one or more parts of the trigeminal nerve. As it becomes chronic, it can seriously affect the quality of life of most patients, and it is expected to increase in incidence in modern aging society. The objective of this systematic review protocol is to provide methods for evaluating the effectiveness and safety of herbal medicines for idiopathic trigeminal neuralgia (ITN).

**Methods:**

A total of 14 databases will be searched for studies uploaded from inception to the present date that investigated the treatment of ITN. These databases are MEDLINE, EMBASE, AMED, Cumulative Index to Nursing and Allied Health Literature (CINAHL), Cochrane Library, PsycARTICLES, four Korean databases, two Chinese databases, and two Japanese databases. We will include randomized controlled trials (RCTs) assessing herbal medicine decoctions used to treat any type of ITN. All RCTs of decoctions or modified decoctions with any type of form of herbal medicine will be eligible for inclusion. The methodological quality of *randomized controlled trials* will be analyzed using the Cochrane Collaboration tool to assess risk of bias, and the confidence in the cumulative evidence will be assessed using the Grading of Recommendations Assessment, Development and Evaluation (GRADE) instrument.

**Ethics and dissemination:**

The results of this systematic review will be published in a peer-reviewed journal and disseminated electronically and in print. To inform and guide healthcare practices, the review will be updated.

**Trial registration number:**

PROSPERO CRD42020129667.

## 1 Introduction

Trigeminal neuralgia (TN), an orofacial pain limited to one or more divisions of the trigeminal nerve, affects one side of the face, except for TN caused by multiple sclerosis. The symptoms of pain in TN are abrupt in onset and usually last for a few seconds. Although patients may report that the symptoms occur spontaneously, these pain paroxysms are always caused by innocuous mechanical stimulation or movement. In 2016, the American Academy of Neurology recognized the shortcomings of the existing classification for TN and developed a new TN classification and diagnostic grading system classified in 3 etiologic categories that accommodates the needs of clinical practice and research, matches the grading system for neuropathic pain, and corresponds to the general pathology of neurological disorders. The three categories are classified as idiopathic trigeminal neuralgia (ITN) that occurs without apparent cause, other classical TN caused by vascular compression of the trigeminal nerve root, and secondary TN, which is the consequence of a major neurologic disease such as multiple sclerosis or tumor of the cerebellopontine angle.^[1]^ The frequency, duration, and severity of the painful attacks of TN gradually increase and frequently become resistant to medication.^[2]^ Thus, neuralgia becomes chronic and can seriously influence the quality of life in the majority of patients with TN and cause cognitive disturbances, such as anxiety and depression.^[3]^

The annual incidence of TN is ∼4.27 per 100,000 population, and TN affects women more often than men.^[4]^ The risk of developing TN is also known to increase with age. In Korea, the overall lifetime prevalence of TN was 0.15% and also was significantly higher in women than in men, and the number of patients over 50 years of age increased by more than 25% in 2017 compared with 2010. In modern society, there is a high possibility of an increase in the elderly population and negligent accidents because of rapid aging; therefore, it seems that the incidence of TN is highly likely to increase.^[5]^ In order to reduce the side effects and socio-economic burden of TN, appropriate therapeutic strategies and related health care policies should be promptly enforced.

Antiepileptic drugs, including carbamazepine, are the first treatment option for TN, but they often fail to relieve pain and may also cause serious side effects such as cognitive impairment, memory loss, and bone marrow suppression.^[6,7]^ If treatment is poor with medications or side effects occur, surgical treatment will be performed. Among surgical procedures, microvascular decompression (MVD) is considered more suitable because the rate of pain recurrence is the lowest^[8]^; however, MVD has the disadvantage that it is limited in its application to neurovascular collisions in vascular compression of the trigeminal nerve root of young adults or healthy elderly people and that about half of the treatment cases experience repeated pain.^[2,9]^ Because of the limitations and deficiencies in pharmacological and surgical therapy for TN, population-based surveys have reported that people with chronic neurological pain are more likely to try complementary and alternative medicine (CAM) therapies such as herbal treatments.^[10–12]^ For this reason, herbal medicine can be an option for the treatment and management of TN patients as a complementary and alternative method.

This proposed review aims to systematically assess evidence of the therapeutic efficacy and safety of herbal medicines for pain control and symptom management in ITN patients among TN patients from randomized controlled trials (RCTs).

## 2 Methods and analysis

### 1.1 Method

#### 1.1.1 Study registration

This systematic review protocol report complies with the Preferred Reporting Items for Systematic Reviews and Meta-Analyses protocols (PRISMA-P).^[13]^ This protocol was registered in PROSPERO (an international prospective register of systematic reviews) 2020 CRD42020129667 (https://www.crd.york.ac.uk/prospero/display_record.php?ID=CRD42020129667).

#### 1.1.2 Dissemination and ethical approval

This systematic review will be published in a peer-reviewed journal and disseminated electronically and in print. The review will be updated to inform and guide health care practices. As this is a study based on a review of published literature, ethical approval is not required.

### 1.2 Data sources

Databases and search terms were determined through discussion between all authors before the literature searches were executed. The following electronic databases will be searched for studies uploaded from inception to the present date that investigated the treatment of ITN: Medical Literature Analysis and Retrieval System Online (MEDLINE), Excerpta Medica database (EMBASE), Allied and Complementary Medicine Database (AMED), Cumulative Index to Nursing and Allied Health Literature (CINAHL), Cochrane Library, and PsycARTICLES. We will also search four Korean databases (KoreaMed, Oriental Medicine Advanced Searching Integrated System [OASIS], the Korean Studies Information Service System [KISS], and Korean Traditional Knowledge Portal [KTCKP]), two Chinese databases (China National Knowledge Infrastructure [CNKI] and WanFang Data), and two Japanese databases (CiNii and Japanese Institutional Repositories Online [JAIRO]).

### 1.3 Eligibility criteria for considering studies for this review

#### 1.3.1 Types of studies

Prospective RCTs that evaluate the effectiveness of herbal medicine formulas as a treatment for ITN will be included in this review. No language restrictions will be imposed. Non-randomized trials, literature research, animal and cell studies, and quasi-RCTs (methods of allocating participants to a treatment group that are not actually random) will be excluded. Trials including healthy participants will be excluded. No language restrictions will be imposed.

#### 1.3.2 Types of participants

Eligible participants are those who were diagnosed with ITN based on the international classification of headache disorders, 3rd edition of Headache Classification Committee of the International Headache Society, regardless of sex, intensity, frequency, duration of ITN, and country of origin. However, the age will be required to be older than 18. To exclude secondary causes, MRI must be employed.

#### 1.3.3 Types of interventions and controls

Both treatment with herbal medicine alone and concurrent treatment with another therapy will be considered acceptable if only herbal medicine is applied to the intervention group and the other treatment is provided equally to both intervention and control groups. Studies evaluating any type of form of herbal medicine, such as decoctions, tablets, capsules, extracts, powders, or pills, will be eligible for inclusion.

For control groups, we will consider placebo or sham, no interventions, and any type of control intervention compared with herbal medicine.

### 1.4 Outcomes and prioritisation

#### 1.4.1 Primary outcomes

Severity of pain will be measured with any valid scales such as visual analog scale (VAS), verbal rating scale (VRS), short-form McGill Pain Questionnaire (SFMPQ), or Numerical Rating Scale (NRS).

#### 1.4.2 Additional outcomes

1. Quality of life (measured with any available assessments, such as EuroQol Survey [EQ-5D], short-form 36 questionnaire [SF-36], and American Chronic Pain Association ranging from 0 [nonfunctioning] to 10 [normal daily activities])
2. Recurrence rate
3. Adverse effects (relevant symptoms caused by herbal medicine)

### 1.5 Data collection and synthesis

#### 1.5.1 Data extraction

A hard copy of every article will be obtained and read in full. Two authors will perform the data extraction and quality assessment using a predefined data extraction form. All authors will conduct the data extraction with a recognized data extraction form founded by all reviewers that includes author, age, country, year of publication, characteristics of participants, intervention, randomized method, blinding, control treatment, main outcomes, and adverse events.

#### 1.5.2 Assessment of risk of bias in individual studies

Risk of bias will be assessed using the Cochrane Handbook risk of bias assessment tool version 5.1.0, which takes into account random sequence generation, allocation concealment, blinding of participants and personnel, blinding of outcome assessment, incomplete outcome data, selective reporting, and other sources of bias. The results of the assessments will be presented using the scores of “L” indicating a low risk of bias, “U” indicating an uncertain risk of bias, and “H” indicating a high risk of bias. Any disagreement will be resolved through discussion among all authors.

#### 1.5.3 Data synthesis

Differences between intervention and control groups will be assessed. Mean differences (MDs) with 95% confidence intervals (CIs) will be used to measure the effects of treatment for continuous data. We will convert other forms of data into MDs. For outcome variables on different scales, we will use standard MDs with 95% CIs. For dichotomous data, we will present treatment effects as relative risks (RRs) with 95% CIs; other binary data will be converted into RR values. For outcome variables with different scales, standardized MD will be used instead of weighted MD.

All statistical analyses will be conducted using Cochrane Collaboration's software program Review Manager version 5.3 (Copenhagen, The Nordic Cochrane Centre, the Cochrane Collaboration, 2014) for Windows. We will contact the corresponding authors of studies with missing information to acquire and verify the data whenever possible. When appropriate, we will pool the data across studies to conduct a meta-analysis using fixed or random effects. We will use GRADEpro software from Cochrane Systematic Reviews to create a Summary of Findings table.

When we detect heterogeneity (defined by results of tests of heterogeneity that indicate *P* < .1 via chi-square tests and Higgins *I*^2^ ≥ 50%), subgroup analyses will be performed to find the cause of the possible causes.^[14]^

## 3 Discussion

TN is known as a neuropathic pain condition affecting the 5th cranial nerve (trigeminal nerve), which is one of the most widely distributed nerves in the head.^[15]^ In traditional medicine, TN has been recognized as a symptom of an invasion of the face because of cold wind pathogens from the outside or unstable body energy caused by excessive stress. In traditional medicine in East Asian countries, various treatment methods including herbal medicine, acupuncture, electroacupuncture, moxibustion, pharmacopuncture, and cupping therapy are frequently used to control and manage neuropathic pain.^[16]^

It has been suggested that people with neuropathic pain are likely to seek alternative methods of pain relief, such as herbal products, because of side effects caused by current pharmacological agents used in the treatment of neuropathic pain.^[17]^ In addition, population-based surveys^[10–12]^ have reported that people with chronic neurological pain are more likely to try CAM therapies such as herbal treatments.

There is evidence that acupuncture is favorable in treating neuropathic pain.^[18,19]^ Although the use of herbal products/preparations for the treatment of neuropathic pain appears promising, there have been reports that stronger evidence is needed for the use of herbal products^[17]^ and that there are insufficient studies on the therapeutic effect of herbal medicines on TN.

Therefore, the authors would like to conduct a systematic review of the impact of herbal medicine therapy on the treatment and management of ITN.

The evidence obtained will provide useful information for patients, practitioners, and health policy makers; and patients suffering from ITN will be able to receive appropriate herbal treatments while doctors will be able to confirm the basis for treatment decisions. From a policy viewpoint, outcomes of this study are also expected to provide basic information for determining the health insurance coverage for herbal medicine and to be used as evidence for establishing an integrated model of East–West treatment for ITN.

Regional differences in the study of herbal remedies in Eastern and Western countries may be considered as a limitation in this systematic review study.

## Author contributions

JHJ and JK conceived the study, developed the criteria, searched the literature, and analyzed the data. JHH wrote the protocol and JHJ and JK revised the manuscript. All authors have read and approved the final manuscript.
